# Characterising the design and methods of continuous glucose monitoring used in behavioural interventions to inform future research in prediabetes

**DOI:** 10.1016/j.landig.2025.100904

**Published:** 2025-11-03

**Authors:** David S Black, Alaina P Vidmar, Braden Barnett

**Affiliations:** Population and Public Health Sciences, Keck School of Medicine of University of Southern California, Los Angeles, CA, USA; Division of Pediatric Endocrinology, Diabetes, and Metabolism, Children’s Hospital Los Angeles, Los Angeles, CA, USA; Division of Endocrinology and Diabetes, Keck School of Medicine of University of Southern California, Los Angeles, CA, USA

## Abstract

Digital health feedback technologies are expected to help address the projected 630 million individuals with prediabetes worldwide by 2045. This Viewpoint article characterises the historical use of continuous glucose monitoring (CGM) systems in behavioural research with a focus on the prediabetic population. We identified 19 peer-reviewed studies through a pragmatic literature review and reported key methodological features, including study design, sensor wear protocols, data masking strategies, the role of CGM in behavioural interventions, and approaches to generate CGM metrics. Based on our literature review, we propose four directions to advance CGM in behavioural intervention research in prediabetes: refining sampling strategies to focus recruitment on individuals with prediabetes to better understand metrics in this population; improving transparency in CGM feedback delivery protocols; reporting a comprehensive and targeted set of CGM metrics; and articulating principles that account for the effects of CGM use within behavioural interventions. This methodological characterisation of CGM is a starting point to enhance research quality and behavioural intervention effectiveness, particularly when integrating CGM systems aimed at supporting dietary, physical activity, or lifestyle modifications among people with prediabetes.

## Introduction

Prediabetes is a metabolic condition linked with the development of diabetes.^1^ The projected 630 million people with prediabetes globally by 2045, along with associated morbidity and mortality,^2,3^ highlights the need for effective behavioural interventions, which are central to targeted diabetes prevention. A major challenge in designing behavioural interventions to prevent chronic diseases, such as type 2 diabetes, lies in ensuring that the outcomes of behavioural changes are observable, timely, and interpretable by individuals at risk. Continuous glucose monitoring (CGM) systems might be capable of addressing this gap by providing real-time, wearable technology that directly measures interstitial glucose concentration and delivers feedback on glycaemic responses resulting from glucose-modifying behaviours, such as diet, physical activity, and other lifestyle modifications.^4^ Previous reviews on this topic have highlighted the potential of CGM in individuals without diabetes,^5^ and evolving CGM metric benchmarks for people with prediabetes enable more precise clinical interpretation.^6,7^ To advance this domain of research, we aimed to characterise the methodological applications of CGM in behavioural intervention research among people with prediabetes.

## Characterising CGM use in behavioural interventions

The table summarises 19 independent human trials that used a CGM system in behavioural interventions for participants with prediabetes. Seven of these trials used a between-groups design. The remaining 12 trials used a within-subjects design. Of these within-subject trials, seven used random sequencing of conditions, whereas five did not. Sample sizes across the trials varied widely, ranging from 3 to 226 participants with prediabetes. The low number of participants in some studies was attributed to several trials involving participants other than those with prediabetes, such as those with normoglycaemia or diabetes along with prediabetes, as indicated in the table.

CGM systems used in the trials were manufactured by three companies: Abbott Libre (n=9), Medtronic (n=6), and Dexcom (n=3). CGM sensor wear durations varied widely, ranging from 2 days to 6 months. Eight trials had a consecutive sensor wear time less than 7 days. Unmasking of CGM feedback was implemented in only ten trials, indicating that just over half of the studies offered glucose feedback to participants. Although the reasons for masking were not commonly specified, masking was used in some cases to minimise the influence of CGM feedback on participant behaviour and glucose concentration, particularly when the primary focus was on a primary diet or physical activity alteration. In many instances, CGM was used solely as a measurement device rather than as a feedback system, including in trials involving acute treadmill exercise or controlled meal laboratory tests.

Major behavioural interventions included dietary modification (11 trials), exercise and lifestyle modification (seven trials), and glucose self-monitoring or goal setting (six trials). Among trials involving glucose self-monitoring, glucose profiles were given to participants as feedback to inform self-management of future glucose concentration through dietary, physical activity, and lifestyle changes. This feedback aided in planning behavioural modifications or to integrate data records with app-based algorithmic models for personalised guidance in relation to glucose control goals. Glucose feedback was provided either by a CGM system app with or without real-time data or by a multiday aggregated profile generated by the research team.

In the analysis of CGM data, one study did not report any CGM-derived variables. Three studies assessed behavioural factors associated with CGM system use, such as perceived usefulness, overall satisfaction, and frequency of self-monitoring. Four other studies analysed one or more variables generated directly from the CGM system as part of the ambulatory glucose profile metrics. These metrics included time-in-range, continuous overall net glycaemic action, and mean amplitude of glycaemic excursions (MAGE). Six studies included CGM metrics in relation to postprandial events. Eight studies included at least one descriptive analysis according to the suggested criteria of glucose concentrations of more than 140 mg/dL to define high, out-of-range glucose concentrations in participants with prediabetes.^6^

## Priorities for future behavioural research using CGM among people with prediabetes

The identified behavioural intervention trials (n=19) using CGM systems among individuals with prediabetes highlight various challenges to be addressed in future research. CGM use in behavioural research appears highly promising because of its ability to provide timely, continuous feedback in natural settings of daily living, yet its use in trials among people with prediabetes remains notably underdeveloped compared with people with diabetes.^27,28^ Based on our literature review, we propose four key directions for advancing the next phase of CGM use in behavioural intervention research in prediabetes: (1) refining sampling strategies to target recruitment of people with prediabetes; (2) improving reporting transparency in CGM feedback delivery; (3) standardising the reporting of CGM metrics highly relevant to prediabetes; and (4) articulating behavioural principles that account for the effect of CGM in interventions for people with prediabetes. Recommendations for future reporting are provided in the panel.

First, there is a need for precise recruitment of individuals meeting established diagnostic criteria for prediabetes, such as fasting glucose concentrations of 100–125 mg/dL, 2 h glucose concentrations of 140–199 mg/dL after a 75 g oral glucose load, or HbA1c concentrations of 5·7–6·4%.^29^ The literature we reviewed comprised heterogeneous samples, encompassing individuals with normoglycaemia, prediabetes, diabetes, or those who are overweight and have obesity, which limits the focus on prediabetes and complicates the rationale for offering CGM feedback in its various forms (eg, use of alarms and setting out-of-range values). For example, in one of the largest trials with 665 participants, only 25 participants had prediabetes.^25^ This heterogeneity in previous samples and the small proportion with prediabetes underscores the importance of targeting populations with prediabetes, as CGM feedback might elicit distinct behavioural responses in individuals with and without diabetes. Focusing future research trials on populations with prediabetes might yield more homogeneous data and enable the development of tailored interventions for this population, which is not yet well engaged with medical care systems based on our clinical experience. For people diagnosed with diabetes, there is an overarching care engagement that might differentially affect adherence to CGM use and behavioural change in response to feedback. Furthermore, more nuanced inclusion criteria, incorporating metabolic phenotyping (eg, insulin resistance via homoeostatic model assessment for insulin resistance or pancreatic beta-cell function via C peptide concentrations), could help with stratifying participants and identifying those most likely to benefit from CGM feedback.

A second, recurring issue that we identified was the absence or scarcity of detail in protocols concerning the CGM feedback supplied to participants, a crucial intervention component with possibly dynamic effects. For example, alarm and visual feedback varies in strength and can impact continuous engagement with feedback and attempts at behaviour change. To clearly understand CGM’s function as an independent variable, researchers could provide protocols that include details of the type, frequency, and timing of feedback participants engage with, ideally supplemented by app usage data. Some CGM apps do not capture use data, and therefore, additional self-reported use assessment might be needed. In studies with unmasked phases, clear instructions on how participants should engage with feedback are needed. For instance, researchers can specify whether participants were instructed to check feedback at predetermined times (eg, before and after meals, hourly, once daily, or every 4 h) or in response to specific out-of-range alert events. Moreover, describing built-in alarm thresholds (eg, 140 *vs* 180 mg/dL) or the silencing of alarms, such as for low glucose values, is crucial to ensure replicability in the delivery of the CGM method. It is also possible that alarms are aversive and thus reduce engagement among people without diabetes. The timing of feedback delivery—immediate and continuous versus delayed and aggregated—might have differential effects on self-controlled behavioural modifications, particularly when combined with other intervention components, such as goal setting or self-monitoring. We observed that some studies aggregated glucose concentrations over long wear periods; it is important to know if this method is inferior to continuous feedback, which can perhaps be susceptible to eliciting false identification of causal predictors (eg, warm *vs* cold temperature causing glucose spikes when a third variable such as juice drinking on hot days was the culprit). Such interpretations were previously identified in a qualitative analysis of adults with prediabetes who wore unmasked CGM for 20 days.^30^

A third issue was the substantial variability in the CGM metrics analysed and reported in studies involving people with prediabetes. Although some consistency was observed in metrics such as mean blood glucose, time-out-of-range, and MAGE, reporting practices remain heterogeneous, highlighting the need for standardised reporting of the complete CGM panel (perhaps as supplemental material) to enhance consistency and comparability across studies when sampling people with prediabetes. Some published recommendations emphasise the inclusion of early dysglycaemic metrics specific to prediabetes, such as maintaining time-in-range (70–140 mg/dL) for more than 95% of the day and the use of glycaemic variability metrics such as SD, coefficient of variation, and MAGE.^6,31^ These metrics quantify glycaemic excursion dynamics, which are particularly relevant for preventing and intervening during early dysglycaemia. Standardising CGM reporting would also benefit future meta-analyses and contribute to a highly cohesive understanding of glycaemic patterns in prediabetes, thus supporting robust evidence gathering for behavioural interventions and expectations for magnitude of change. However, including contextual information on how participants are instructed to interpret these metrics, such as time-in-range, and whether they were aware of potential sources of variability (eg, exercise and meal timing) are equally important, as such data will be essential for understanding the mechanisms of behavioural change to be attributed to the observed changes in glucose panel scores.^32^ Moreover, the use of personalised glycaemic targets based on individual factors such as baseline glucose concentrations or insulin sensitivity might further optimise intervention strategies.^33^

Finally, less attention has been given to the principles underlying the function of CGM in behavioural sciences. CGM is a metric-quantifying system, but it is unclear how it functions on the human organism in terms of changing behaviour. The diverse range of intervention strategies used in the studies we reviewed illustrates the flexibility of CGM. Some interventions used it solely for data collection, whereas others integrated CGM feedback as part of a multicomponent approach, including dietary, exercise, lifestyle, and self-monitoring components. However, key questions remain about why CGM feedback modifies glucose concentrations.^7^ For instance, whether CGM functions by contingency-shaped problem solving or by challenging attitudes and beliefs about some foods or other core principles is unclear. Even the use of standalone CGM for glycaemic control in prediabetes remains unclear in principle, as its use has often been incorporated into broader interventions without evaluating its singular effects. To maximise CGM’s utility, it is important to clarify these distinctions and better articulate the behavioural principles guiding its use, particularly through concepts such as contingency-shaped problem solving given that the power of feedback rests on the individual’s ability to interpret that feedback accurately and discriminate between behaviours that generated differences in values.^30^ With coinciding advancements in other wearable technologies such as smartwatches and smart rings, behavioural interventions can be strengthened by revealing the degree to which changes in response (eg, step count immediately after a meal) can directly reveal a reduction in glycaemic excursion. For instance, increased step count improves glucose control and consequently, the CGM feedback can function as a positive reinforcer for step count and related physical activity.

Originally developed for diabetes self-management, CGM is a glucose measurement tool and feedback generator for self-monitoring. As a tool generating useful information, it offers a unique opportunity to advance behavioural intervention research in prediabetes. By addressing the many challenges identified in our Viewpoint, future studies can better leverage CGM systems to generate actionable insights, optimise intervention strategies, and possibly prevent type 2 diabetes in the population with prediabetes.^1–9,25–33^

## Figures and Tables

**Table:** Summary of behavioural interventions using a CGM system among adults with prediabetes, n=19 studies

	Study design	CGM wear protocol	CGM masked	PreD N	CGM system use in relation to the behavioural intervention	CGM metrics
Ahn et al (2023)^8^	RCT, 2 groups	CGM (Abbott Libre) for all 4 weeks of INT	No	Mixed <45*	CGM feedback displayed to wearer on device alongside a dietary INT; display showed current glucose level, glucose trend, and 8 h aggregated glucose; wearer instructed on a glycaemic index target according to their height and weight	None
Ahn et al (2024)^9^	WS, 3 conditions	CGM (Dexcom first gen) for 1 week BL, 2 weeks during, and 1 week after INT	No	9	CGM feedback displayed to wearer on device alongside an exercise INT; display showed mean glucose level, hypoglycaemia, and hyperglycaemia; wearer instructed to report aggregated glucose levels weekly	MBG, TOR†, postprandial BG
Bailey et al (2016)^10^	RCT, 2 groups	CGM (Medtronic MiniMed) for 5 days during 1 week, 4 weeks, and 7 weeks of INT	No	3	CGM feedback displayed to wearer on device alongside an exercise INT; display showed current glucose levels and a graph for past 24 h aggregated glucose; wearer instructed to monitor glucose levels 4 times a day and instructed to set goals according to their personal glucose graph	N times of self-monitored glucose levels
Ben-Yacov et al (2021)^11^	RCT, 2 groups	CGM (Abbott Libre) for 2 weeks at BL, 6 months of intervention, and 2 weeks at 9 months and 12 months FU	Yes	225	CGM feedback masked from wearer alongside a dietary INT; display feature not used; CGM was for measurement only	MBG, TOR†
Bruno et al (2024)^12^	RCT, 2 groups	CGM (Abbott Libre) for 2 weeks of study period	Yes	10	CGM feedback masked from wearer alongside a dietary INT; display feature not used; CGM was for measurement only	MBG, SD, MAGE, TIR, TOR†
DiPietro et al (2013)^13^	WS, 3 RS with WO	CGM (Metronic MiniMed) for 48 h at three visits, which were 4 weeks apart	Yes	10	CGM feedback masked from wearer alongside an exercise INT; display feature not used; CGM was for measurement only	MBG, postprandial BG, AUC
Faerch et al (2021)^14^	RCT, 4 groups	CGM (Medtronic iPro2) for 6 days at BL, 6 weeks and 13 weeks of INT, and 26 weeks of FU	Yes	120	CGM feedback masked from wearer alongside exercise and medication INTs; display feature not used; CGM was for measurement only	GV, MBG, MAGE, TOR†, SD, CV
Francois et al (2014)^15^	WS, 3 RS with WO	CGM (Medtronic iPro2) for 5 days including BL, during INT, and day after INT	Yes	9	CGM feedback masked from wearer alongside an exercise INT; display feature not used; CGM was for measurement only	MBG, postprandial BG, AUC
Gardner et al (2022)^16^	WS, 2 RS	CGM (Dexcom G6) for 2 weeks at BL; 4 weeks, 12 weeks, 16 weeks, and 24 weeks of FU	No	23	CGM feedback displayed to wearer on device alongside a dietary INT; display showed current glucose levels; wearer instructions not stated	MBG, TIR, CV
Gay et al (2018)^17^	WS, 3 RS	CGM (Medtronic iPro2) for 4 days of study	Yes	9	CGM feedback masked from wearer alongside an exercise INT; display feature not used; CGM was for measurement only	MBG, AUC, postprandial AUC
Gravesteijn et al (2023)^18^	WS, 2 RS with WO	CGM (Abbott Libre) for 48 h in the last week of BL and during INT	Yes	33	CGM feedback masked from wearer alongside a dietary INT; display feature not used; CGM was for measurement only	MBG, AUC
Gulati et al (2023)^19^	WS, 2 RS with WO	CGM (Medtronic iPro2) for 3 days of INT and control period	Yes	60	CGM worn alongside a dietary INT; CGM was for measurement only	MBG, TIR, TOR†, AUC, GV, MAGE, SD, MoDD
Kharmats et al (2023)^20^	RCT, 2 groups	CGM (Abbott Libre) for 14 days at BL and at 3 months and 6 months	No	Mixed <156*	CGM feedback masked from wearer on device alongside a dietary INT with weight loss support; display feature not used; CGM was for measurement only	MAGE
Luo et al (2022)^21^	RCT, 3 groups	CGM (Abbott Libre) for 14 days at BL, and 3 months and 6 months during INT	No	226	CGM feedback displayed to wearer on device alongside a dietary INT; display feature not used; CGM was for measurement only	TOR, SD
Parr et al (2018)^22^	WS, 2 RS with WO	CGM (Medtronic iPro2) for 48 h at the end of each condition	No	13	CGM feedback displayed to wearer on device alongside a dietary INT; display feature not described; wearer instructions not stated	MBG, postprandial AUC, MAGE, CONGA, SD
Richardson et al (2024)^23^	WS, 2 conditions	CGM (Dexcom G6) for 10 days masked BL and 10 days during INT	Yes	25	CGM feedback first masked from wearer at baseline then displayed to wearer on device alongside NDPP-recommended lifestyle INT; display showed glucose levels and excursions; wearer instructed on methods to limit glucose excursions >140 mg/dL	Sensor acceptability
Yost et al (2020)^24^	WS, 2 conditions	CGM (Abbott Libre) for 10 days masked BL then 10 days during INT	Yes	15	CGM feedback first masked from wearer then displayed to wearer alongside a dietary INT; unmasked phase; wearer instructed to compare their food logs with a printout of their glucose levels	MBG, TAR†, sensor satisfaction
Zahedani et al (2021)^25^	WS, 1 condition	CGM (Abbott Libre) for 10 days	No	Mixed 25	CGM feedback displayed to wearer on device alongside a mobile health app INT; display showed glucose values after scanning for up to 8 h of data (ie, Flash CGM), and no alarms were used; wearer instructed to consume three different meals and identify glycaemia patterns	TIR†, postprandial BG
Zahedani et al (2023)^26^	WS, 2 conditions	CGM (Abbott Libre) for 28 days	No	206	CGM feedback displayed to wearer on device alongside a digital health app INT; display showed machine learning-based predictions of glucose mapped onto the personal food and activity logs; wearer instructed by app on behaviour modification activities to control future glucose spikes	TIR†, GMI

AUC=area under the curve. BG=blood glucose. BL=baseline. CGM=continuous glucose monitoring. CONGA=continuous overlapping net glycaemic action. CV=coefficient of variation. FU=follow-up. GMI=glucose management indicator—medication treatments, medical interventions such as surgery, or pill or capsule supplementation not included as behavioural interventions. GV=glucose variability. INT=intervention. MAGE=mean amplitude of glycaemic excursions. MBG=mean blood glucose. MoDD=mean of daily differences. NDPP=National Diabetes Prevention Program. PreD N=number of participants in the sample with self-reported or measure-determined prediabetes status; some samples were mixed with normoglycemic, prediabetes (PreD), and diabetes (N indicates the subsample with only PreD). RCT=randomised controlled trial. RS=randomly sequenced conditions. SD=standard deviation in relation to the complete metric profile indicated in the column header. TAR=time above range. TBR=time below range. TIR=time in range. TOR=time outside range. WO=washout period between conditions. WS=within-subject design.

* Study did not indicate N for PreD, and hence, this number should be less than the sample sizes of 45 and 156.

† The guideline for glucose level >140 mg/dL for prediabetes was analysed.

## Search strategy and selection criteria

We performed a pragmatic literature review to identify published articles on behavioural interventions using continuous glucose monitoring (CGM) among people with prediabetes. DSB formulated the research question, and both DSB and APV identified and selected relevant studies through an iterative process. DSB, APV, and BB summarised the results. DSB developed the initial search strategy using a combination of MeSH and free-text keywords. The search was done on July 10, 2024, restricted to studies published since 2000, involving human participants, and written in English. We searched Google Scholar, PubMed, and Web of Science using the search terms “continuous glucose monitoring” OR “CGM” AND “prediabetes” OR “insulin resistance”. No additional efforts were made to identify grey literature, including other study registries, websites, or conference proceedings. On Dec 6, 2024, APV repeated the search in the same bibliographic databases to identify any more recent studies.

Retrieved titles and abstracts were initially screened by DSB, followed by full text reviews by DSB and APV. 509 abstracts were screened, and 59 full text articles were reviewed. Of these full text articles, 41 were excluded for the following reasons: absence of CGM use, no behavioural intervention, or non-adult or non-prediabetes samples.

Inclusion criteria for the initial screening of abstracts were articles written in English, inclusion of adult participants with prediabetes, reporting of behavioural intervention (medical interventions such as surgery, medication, or treatments using capsules or pills were excluded), and inclusion of a CGM wear period. Studies limited to participants with type 1 or type 2 diabetes were excluded. For the subsequent full text review, inclusion criteria were full text relevance to the research question and peer-reviewed publication. Dissertations, conference abstracts and proceedings, unpublished protocols, and commentaries or opinion pieces were excluded.

## *Panel:* Guidelines on reporting CGM methods in behavioural intervention research in prediabetes to generate opportunities for future replication

**Sampling:** Describe participant eligibility for prediabetes based on clinical or biometric criteria, including:

- Fasting glucose concentration (100–125 mg/dL)
- 2 h glucose concentration of 140–199 mg/dL after a 75 g oral glucose load
- HbA1c concentration (5·7–6·4%)
- CGM-derived estimated metric such as sensor estimated HbA1c (eA1c)
- Insulin resistance (fasting homoeostatic model assessment of insulin resistance)
- Pancreatic beta-cell function (blood or urine C-peptide concentration)

**User protocol:** Detail the structure and timing of participant exposure to CGM feedback, including:

- Manufacturer CGM system and version used
- Instructed frequency of self-monitoring of CGM feedback (eg, review glucose concentrations every 4 h, 20 min after meals, and ad libitum)
- Type and timing of glucose concentration feedback in relation to intervention content (eg, current glucose concentration line graphic tracings with values added every 5 min; numerical values only)
- Set alarm thresholds or silence alerts and alarms (eg, high alarms set at 140 or 180 mg/dL, turning off alerts during established sleep hours from 22:00 h to 06:00 h, and low alarms turned off)
- Use of other technologies (eg, accelerometer and smart rings) and rationale for use in relation to the CGM system

**Metrics:** Describe how CGM data were prepared and quantified, including:

- Analysis app and version
- Data capture metrics (eg, median wear time with observed range and percentage of sample wear time)
- Target metrics based on the study hypothesis (eg, mean glucose concentrations, time-in-range, standard deviation of the metric, mean amplitude of glucose excursion) and data trimming description to account for calibration periods
- Reporting of full glucose panel metrics as supplementary material in publications or data repositories to enable future analysis
- Time period for target metrics and full panel metrics (eg, analytical sample had a minimum of 7 continuous days of data; there was a gap of 7–13 days between the end of the first sensor wear period and start of the second sensor wear period)

**Intervention:** Specify CGM use in relation to the intervention’s theoretical and behavioural mechanisms, including:

- Rationale for using CGM in the selected intervention (eg, informing dietary change, self-monitoring, and measurement)
- Key principles relating CGM to the intervention (eg, contingency-shaped problem solving, contingency management, reinforcement schedules, goal orienting, and stimulus response)
- Control groups that account for standalone effect of unmasked CGM to clarify the effect of the selected intervention beyond that of CGM feedback
- Measured variables likely responsible for causing the changes in CGM panel metrics (eg, dietary and physical activity logs and app engagement)

CGM=continuous glucose monitoring.
